# Action-related brain dynamics during leader–follower coordination

**DOI:** 10.64898/2026.10.11.758328

**Published:** 2026-10-18

**Authors:** Santeri Ruuskanen, Juan Camilo Avendano-Diaz, Riitta Hari, Lauri Parkkonen

## Abstract

Leader–follower dynamics are essential for social interaction, enabling smooth inter-personal coordination. Although neuroimaging studies have identified brain networks associated with leading and following, the dynamical brain signatures supporting these roles remain poorly characterized. Here, sixteen dyads performed a joint-improvisation mirror-game task with predefined leader–follower roles while magnetoencephalography (MEG) and hand kinematics (acceleration) were simultaneously recorded. We investigated role-specific modulations of source-space MEG activity in delta (0.5–4 Hz), theta (4–8 Hz), alpha (8–13 Hz), beta (13–30 Hz), and gamma (40–90 Hz) frequency bands, as well as coherence between cortical activity and movement kinematics. Compared with leaders, followers exhibited stronger theta-band activity in a cluster comprising frontal, cingulate, and sensorimotor regions, and stronger coherence between movement kinematics and occipital cortical activity, consistent with enhanced monitoring, adaptation, and reliance on visual kinematic information. In contrast, leaders showed reduced alpha-band power in sensorimotor and premotor regions, and stronger beta-band activity in occipital cortices, likely reflecting greater demands for action planning and predictive control. Our results identify complementary spectral and action-related brain signatures associated with leader–follower coordination during joint improvisation, providing new insights into the neural underpinnings of real-time interpersonal coordination.

